# Combining computer vision and deep learning to enable ultra-scale aerial phenotyping and precision agriculture: A case study of lettuce production

**DOI:** 10.1038/s41438-019-0151-5

**Published:** 2019-06-01

**Authors:** Alan Bauer, Aaron George Bostrom, Joshua Ball, Christopher Applegate, Tao Cheng, Stephen Laycock, Sergio Moreno Rojas, Jacob Kirwan, Ji Zhou

**Affiliations:** 1 Earlham Institute, Norwich Research Park, Norwich, NR4 7UZ UK; 2 Plant Phenomics Research Center, China-UK Plant Phenomics Research Centre, Nanjing Agricultural University, Nanjing, 210095 Jiangsu China; 3 School of Computing Sciences, University of East Anglia, Norwich Research Park, Norwich, NR4 7TJ UK; 4 National Engineering and Technology Center for Information Agriculture, MARA Key Laboratory for Crop System Analysis and Decision Making, Jiangsu Key Laboratory for Information Agriculture, Nanjing Agricultural University, Nanjing, 210095 Jiangsu China; 5 G’s Growers Limited, Ely, Cambridgeshire CB7 5TZ UK

**Keywords:** Field trials, Agricultural genetics, High-throughput screening

## Abstract

Aerial imagery is regularly used by crop researchers, growers and farmers to monitor crops during the growing season. To extract meaningful information from large-scale aerial images collected from the field, high-throughput phenotypic analysis solutions are required, which not only produce high-quality measures of key crop traits, but also support professionals to make prompt and reliable crop management decisions. Here, we report AirSurf, an automated and open-source analytic platform that combines modern computer vision, up-to-date machine learning, and modular software engineering in order to measure yield-related phenotypes from ultra-large aerial imagery. To quantify millions of in-field lettuces acquired by fixed-wing light aircrafts equipped with normalised difference vegetation index (NDVI) sensors, we customised AirSurf by combining computer vision algorithms and a deep-learning classifier trained with over 100,000 labelled lettuce signals. The tailored platform, AirSurf-*Lettuce*, is capable of scoring and categorising iceberg lettuces with high accuracy (>98%). Furthermore, novel analysis functions have been developed to map lettuce size distribution across the field, based on which associated global positioning system (GPS) tagged harvest regions have been identified to enable growers and farmers to conduct precision agricultural practises in order to improve the actual yield as well as crop marketability before the harvest.

## Introduction

As an important source of vitamins, minerals, and trace elements, leaf vegetables play crucial roles in human nutrition^1^. Lettuce (*Lactuca sativa* L.), one of the most popular staple vegetable foods, has a wide range of tastes and textures cultivated for diverse customer needs^2^. Recent research also indicates that lettuce consumption has positive effects on the reduction of cardiovascular disease and chronic conditions due to its rich nutrients such as vitamin A, *Beta-carotene*, folate, and iron content^3^. While lettuce is an important and nutritional crop, fluctuating environments can increase the fragility of its production^4^. For example, the bad weather in Spain in early 2017 led to retail prices of lettuce products nearly tripled in UK supermarkets^5^. Severe weather not only causes supply shortage, but also affects crop quality. According to previous studies on lettuce growth and development^6,7^, young plants at newly planted phase (i.e., from cotyledons unfolded to three true leaves stage) require cool and damp weather after the transplantation from the greenhouse to the field, whereas lettuce leaves can rapidly become bitter and inedible if the growth is accelerated by high ambient temperature at the head maturity phase (i.e., the growth stage before flowering). Because of the dynamic nature of lettuce production, the actual yield of lettuces in commercial operations is only around 70–80% of the planted quantity^8^. Hence, to ensure the consistency of supply and quality, it is important for growers and farmers to closely monitor their crops during key growth stages, so that prompt and reliable crop management decisions can be made under changeable agricultural conditions^9^.

Aerial field phenotyping has become a popular approach for monitoring crops in recent years. Because it can acquire a large number of crop imagery in field experiments using visible, thermal, and multi-spectrum sensors, it has been widely applied to breeding, farming and crop research^10^. To ensure high-quality aerial image acquisition, the flight route and altitude need to be pre-determined together with the selection of appropriate imaging sensors^11^. For example, for physiological traits such as vegetative greenness and canopy structure, a high-definition RGB camera is sufficient; however, many vegetation indices rely on multi- and hyper-spectral imaging sensors to assess important traits such as biomass, stress level, and yield potential^12^. Recently, with the development of image stitching algorithms and orthomosaic generation methods, very detailed crop images can be collected by unmanned aerial vehicles (UAVs) and fixed-wing light aircrafts, which can enable high-quality field phenotyping and high-throughput phenotypic analysis^13^.

To extract meaningful phenotypic information from large-scale image datasets, a variety of computer vision^14^, machine learning (ML), and deep learning (DL) approaches^15^ have been utilised. In recent years, much attention has been paid to ML/DL techniques, based on which computational algorithms and learning models were built to accomplish tasks such as vision-based feature selection, image object classification, and pattern prediction^16–18^. With adequate training data, suitable learning algorithms, and well-defined predictive outcomes, the integration of computer vision, ML/DL, and newly emerged analytic solutions (e.g., distributed computing) could lead to a step change for plant phenomics research in the near future^19^.

In this article, we present a new analytic platform called AirSurf developed for ultra-scale aerial phenotyping and yield-related phenotypic analysis. The software platform is open-source and combines tasks such as normalised difference vegetation index (NDVI) aerial imagery for data collection, computer vision for image processing, deep learning (i.e., convolutional neural networks, CNNs) for crop counting, and supervised machine learning for crop quality assessment. AirSurf was customised for commercial lettuce production so that it could be used to analyse millions of lettuces across the field. We call the tailored software platform “AirSurf-Lettuce” (AirSurf-L), which embeds a CNN model trained with over 100,000 labelled lettuce signals to measure lettuce heads and their plantation layouts based on ultra-large NDVI images. After scoring lettuce, unsupervised ML algorithms were used to classify lettuce heads into three size categories (i.e., small, medium and large) for assessing lettuce quality. To connect phenotypic analysis with marketability and crop management decisions, a novel function has been developed in AirSurf-L to associate global positioning system (GPS) coordinates in a given field with the in-field lettuce size distribution, based on which efficient harvesting strategies could be formed to increase marketable yield.

## Materials and methods

### NDVI aerial imaging and experimental fields

NDVI correlates well with leaf area index and biomass^20^ and hence was chosen for yield-related field phenotyping. The imaging sensor used is an industrial standard camera, as previously described^21^. The aerial imaging was carried out by a ‘Sky Arrow’ light aircraft, the lightest weight class (Very Light Aircraft, VLA) of any commercial aircraft, which allowed the pilot to fly with very little fuel, less than an average farm vehicle. Using VLA at 1000 feet (around 305m) in the sky, vast areas can be covered at a flight speed of 180–200 km/h, during which the NDVI sensor can gather ultra-scale crop imagery to cover four or five fields in a single flight.

**Fig. 1:**
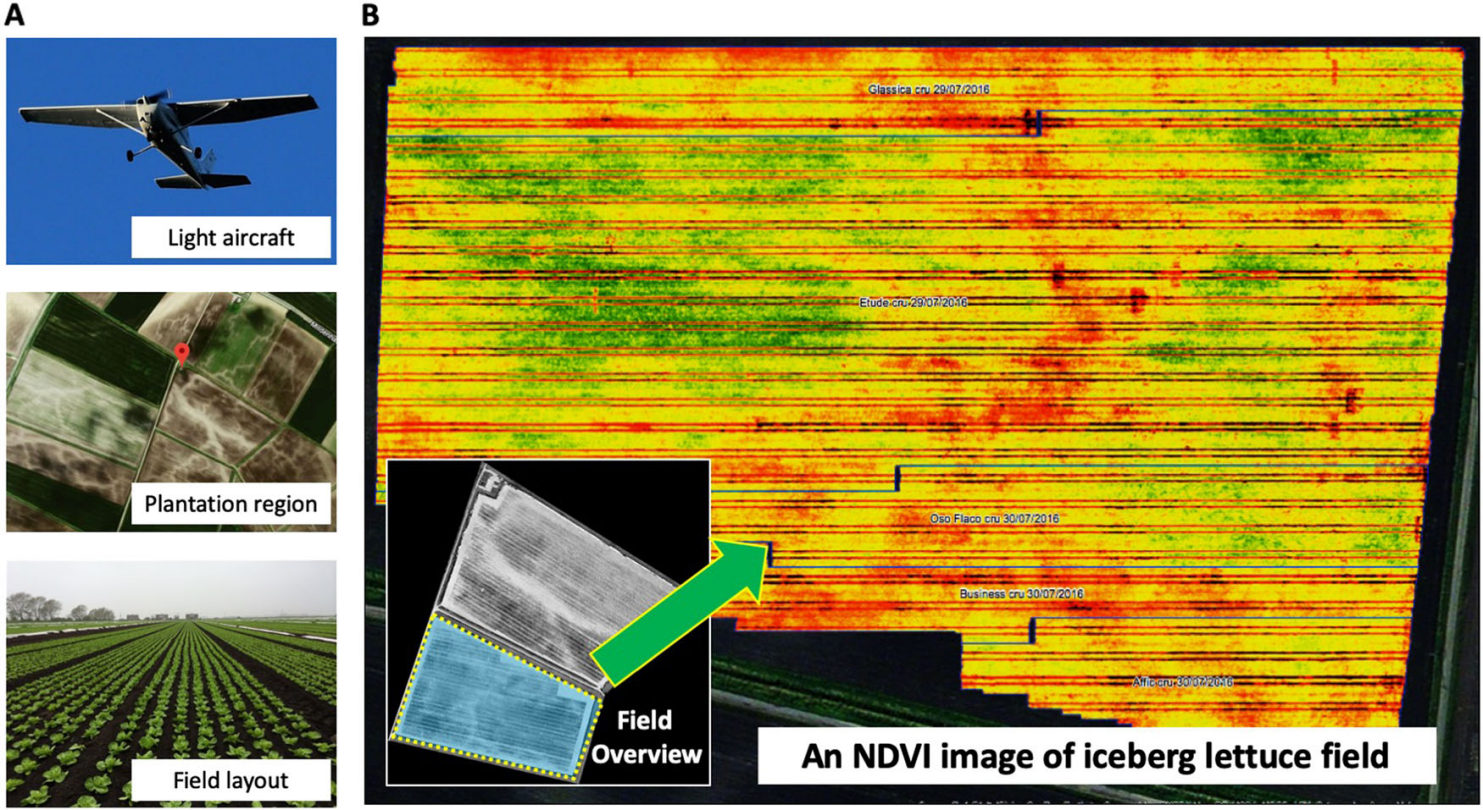
Ultra-scale NDVI aerial imaging accomplished routinely through a fixed-wing light aircraft operated by G’s Growers. **a** The flying route and aerial imaging were designed to facilitate cross-site crop layout assessment and yield prediction. **b** A series of ultra-large NDVI images at 3 cm GSD spatial resolution were acquired to record 0.8–1.6 million lettuce heads per field, at H1 and H2 stages

The ultra-large aerial NDVI imagery was acquired routinely (i.e., four-five times per season) by G’s Growers, the second largest vegetable grower in the UK. The flying route and the imaging protocol were designed to facilitate cross-site crop assessment and yield prediction (Fig. 1a). In this study, we used a series of collected ultra-large NDVI images (1.5–2 GB per image) at 3 cm ground sample distance (GSD) spatial resolution, for iceberg lettuces at H1 and H2 stages (i.e., moderate compact and crushable head), before lettuce leaves were largely overlapped. Experimental fields in the study were all located near Ely, Cambridgeshire UK, ranging from 10 to 20 hectares, with between 800,000 and 1.6 million lettuce heads in a single field. One field (Field A, Fig. 1b) planted with around 1 million lettuce heads was used to explain the analysis workflow and associated algorithms of AirSurf-L in the following sections. A high-level manual yield counting was conducted by G’s growers’ field specialists during the harvest, which was used to verify and improve the platform. Lettuces in subsections randomly selected from Field A were scored manually by laboratory technicians at Norwich Research Park and then used as training datasets for establishing the deep learning model.

### Data construction for training and testing

To generate sound datasets for ML-based image analysis, we randomly selected 60 patches (i.e., subsections) of the field of varying sizes, each containing between 300 and 1000 lettuce heads. We then manually labelled each lettuce in the selected patches with a red dot (Supplementary Fig. 1). Each labelled lettuce, i.e., a red dot, is identified by a 20 × 20 pixel bounding box that can enclose a single lettuce head. We used these bounding boxes, as well as images that did not correspond to lettuce heads, to train a CNN classifier to recognise and separate millions of lettuces in the plantation region. The pixels contained within a bounding box were also used for defining lettuce size. A training dataset with over 100,000 20 × 20 pixel labelled bounding boxes has been created, amongst which 50% are lettuces and the remaining are background signals such as soil, edges of the field, and other non-lettuce objects. Following a standard CNN segmentation approach^22^, we designed a non-overlapping sliding window function to go through the whole field to separate foreground and background signals (i.e., splitting lettuce and non-lettuce objects). Training and testing datasets are equally balanced. Validation sets are used alongside training sets to verify the performance of the model, which can prevent overfitting in model training and allow us to fine-tune hyperparameters of different learning layers^23^.

### The analysis workflow of AirSurf-Lettuce

**Fig. 2:**
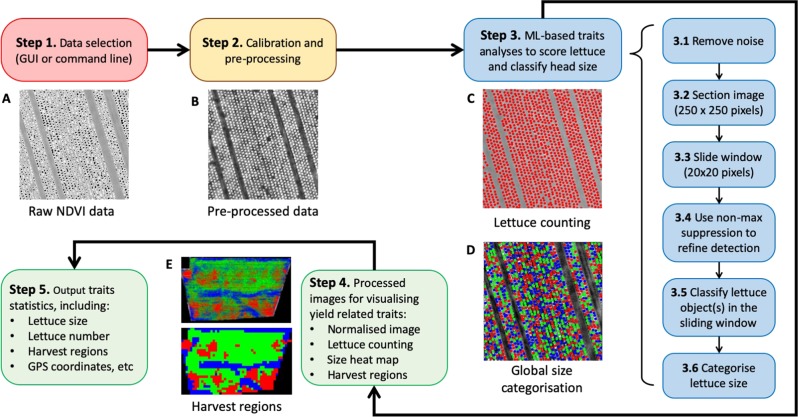
A high-level analysis workflow of AirSurf-Lettuce. **a** Step 1 accepts raw NDVI images as input imagery data (pixels with extremely high NDVI signals are overflowed). **b** Step 2 pre-processes the raw NDVI images to calibrate intensity distribution and correct overflowing pixels. **c**, **d** Step 3 carries out ML-based traits analyses to quantify lettuce number and classify head size in a given NDVI image. **e** Steps 4&5 visualise and export statistics of the traits analyses detection, including yield-related phenotypes such as lettuce counting, size distribution, and harvest regions, and associated GPS coordinates

The analysis of yield-related phenotypes was based on NDVI signals of iceberg lettuces across the field. Figure 2 shows a high-level analysis workflow of AirSurf-L, which consists of five steps: data input, image calibration and pre-processing, ML-based traits analyses, results visualisation, and quantifications of yield-related phenotypes. *Step 1* accepts raw NDVI images as grey-level imagery datasets. As pixels with extremely high NDVI signals usually have overflowed intensity values (i.e., black pixels in Fig. 2a), a pre-processing step (*Step 2*) is designed to calibrate raw NDVI images, so that intensity distribution can be normalised to correct overflowing pixels. At this step, an algorithm called contrast limited adaptive histogram equalisation (CLAHE)^24^ is applied to increase the contrast between the foreground (i.e., lettuces) and background (e.g., soils) in a given NDVI image (Fig. 2b). Supplementary File S1 provides pseudo code and explanations of the image calibration and pre-processing step to ensure high-quality inputs of the learning model.

*Step 3* carries out ML-based traits analyses that quantify lettuce number (Fig. 2c) and classify head size (Fig. 2d). It includes six steps: removing noise signals, partitioning a given image into sections (250 × 250 pixels) for local analysis, producing a sliding window (20 × 20 pixels) to traverse within a sectioned image, using non-max suppression to detect lettuces, and classifying recognised lettuces into three size categorises (i.e., small, medium and large). The analysis result is visualised in *Step 4*, where lettuce counting, size distribution map, and GPS-tagged harvest regions are saved as a series of processed images (Fig. 2e). At the final step (*Step 5*), statistics of yield-related traits are exported to a comma-separated values (CSV) file, including lettuce counts per field, lettuce size distribution, lettuce number and size measures within GPS grids, harvest regions, and associated GPS coordinates (Supplementary File S2). To enable users to carry out the above analysis workflow easily, a graphical user interface (GUI) software application has been developed.

### AirSurf-Lettuce GUI

**Fig. 3:**
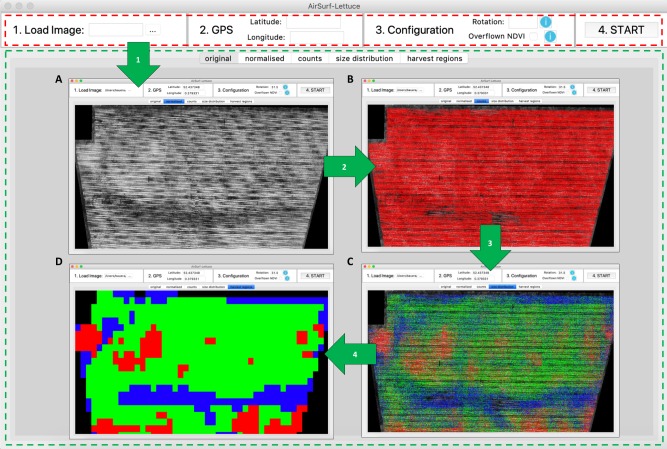
Two sections designed in the AirSurf-Lettuce GUI. **a** A processed image after pre-processing and calibration (in the normalised tab). **b** A processed image after lettuce counting (in the counts tab). **c** A processed image after lettuce size categorisation (in the size distribution tab). **d** A processed image after identifying harvest regions and GPS coordinates (in the harvest regions tab)

The GUI of AirSurf-L (Fig. 3) was developed using the native python GUI package, Tkinter^25^, which allows the software application to be executed on different operating systems such as Windows and Mac OS (note: we only provided a packaged.exe executable file on the GitHub, see availability and requirements). Following the systems design described previously^26^, the GUI uses an easy-to-follow approach to implement the phenotypic analysis workflow. The GUI window is divided into two parts: input section and display section. In the input section (dash rectangle coloured red in Fig. 3), a user needs to firstly load an NDVI image, which will be displayed instantaneously in the display section (dash rectangle coloured green), in the *original* tab. Secondly, the user needs to enter GPS coordinates of the field (i.e., the top left corner of the input image, which can be retrieved from the metadata or Google Maps). Thirdly, the user is required to define the rotation value of the input image (in degrees) in comparison with the north geographical direction, so that GPS calculation can be standardised. Then, the user can tell the software whether the input image contains overflown NDVI signals; if so, an extra calibration process will be triggered (Fig. 2, *Step 2*). Finally, after entering a small number of input parameters, the user can click the Start button to initiate the analysis workflow.

The GUI software follows each step described in the analysis workflow to accomplish automated phenotypic analysis. When a step is finished, an associated processed image will be displayed in the display section, showing the intermediate result of the analysis. Four processed images will be presented in the GUI window: a pre-processed and calibrated image (Fig. 3a, in the *normalised* tab), an image after lettuce scoring (Fig. 3b, in the *counts* tab), an image after size categorisation (Fig. 3c, in the *size distribution* tab), and a processed image after identifying harvest regions and their associated GPS coordinates (Fig. 3d, in the *harvest regions* tab). All processed images are saved in a result folder, along with a CSV file that contains analysis results (Supplementary File S2).

### Neural network architecture

**Fig. 4:**
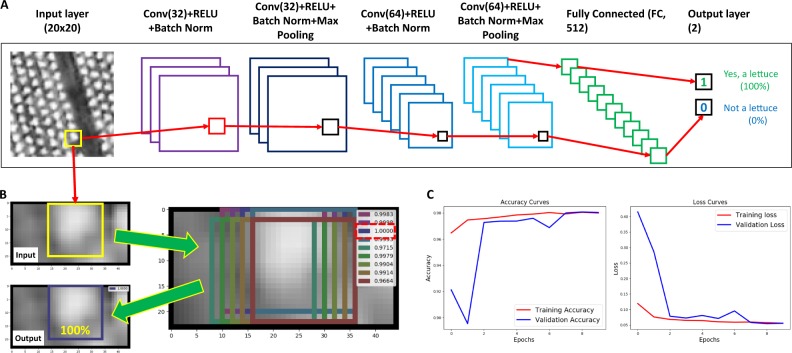
A CNN-based learning architecture established for lettuce counting. **a** The architecture of the trained CNN model, which generates a binary output representing the probability of whether a yellow bounding box contains a lettuce signal. **b** If the probability is close to 100%, it indicates that it is highly likely that the bounding box encloses a whole lettuce. **c** The training and validation accuracy and loss curves of the model

Similar to AlexNet^27^, a CNN-based learning architecture was established using the labelled training datasets. Figure 4a demonstrates the architecture of the CNN model, including (1) a convolutional (Conv2D) layer with 32 filters and a 3 × 3 kernel, with a rectified linear unit (ReLU) as the activation function, and batch normalisation to accelerate the learning process to enable higher learning rates^28^; (2) the same block is then repeated together with a max pooling layer to down-sample input using a 2 × 2 kernel based on the assumption that useful input features could be contained in sub-regions; (3) after that, a second convolutional block is constructed, consisting of a Conv2D layer with 64 filters, a 3 × 3 kernel, a ReLU activation, and batch normalisation; (4) finally, this block is repeated, followed by another max pooling layer (with a 2 × 2 kernel) to complete the learning procedure. After the convolutional layers, learning layers are connected to a fully connected layer of size 512, which is followed by a dropout layer with a 50% chance. To complete the learning architecture, a binary output generates the probability of whether a given bounding box (20 × 20 pixels) contains a lettuce signal. If the probability equals or is close to 100%, it indicates that it is highly likely that the bounding box contains a complete lettuce head (Fig. 4b). The above architecture is commonly applied to vision-based object detection problems^29^. The training and validation accuracy and loss curves are reported in Fig. 4c, showing that the model converges in only 10 epochs. More importantly, to avoid overfitting, the stopping criterion was designed to guarantee the validation accuracy is higher than the training accuracy, ensuring the generalisation of the learning model. To avoid the overfitting issue of our model, the labelled data was also divided equally into train and validation sets when training the model.

The architecture is shallower than AlexNet and other modern deep learning architectures for a number of reasons: (1) the size of our dataset is relatively small for establishing very deep learning networks; (2) our target is a binary classification problem (i.e., whether or not a given bounding box contains a whole lettuce head), different from ImageNet classification tasks; (3) larger and deeper neural networks require more time to train, which can be slower to execute and not feasible for prompt crop management decision required in precision agriculture.

### Size categorisation algorithm

After AirSurf-L identifies bounding boxes containing lettuce heads, we employed an unsupervised ML approach to categorise lettuce into three sizes: small, medium and large. The algorithm can be easily changed to classify more size categories, if required. Pixels in the bounding box region are extracted and then NDVI values of all the pixels are put into bins. The histogram included 10 bins that spread across the NDVI value range (0–255). We included two important aspects when categorising lettuce sizes: (1) lower NDVI surrounding values do not determine the lettuce size; (2) higher NDVI values are more important for the size categorisation. As such, a geometric pattern of NDVI values for each bin was created, i.e., 64, 128, 160, 192, 208, 224, 232, 240, 244, 248, 250, 252, 253, and 254. With these cut-off values, most of the background pixels were captured in the first two bins, along with the increasing weight when values approach 255.

Having transformed the pixel regions into a series of bins, we were able to compare different regions and cluster them into three size groups using k-means clustering with the *k* value set to three. Then, clustering results are sorted through calculating the dot product between the weight vector and the cluster count vector (based on the number of bins). These sorted values determine which clustering result corresponds to which size, which are then applied to each lettuce detected in the field. Three colours are used to indicate size categories: blue for small, green for medium, and red for large (Fig. 3c).

## Results

### Counting lettuces with a CNN classifier

**Fig. 5:**
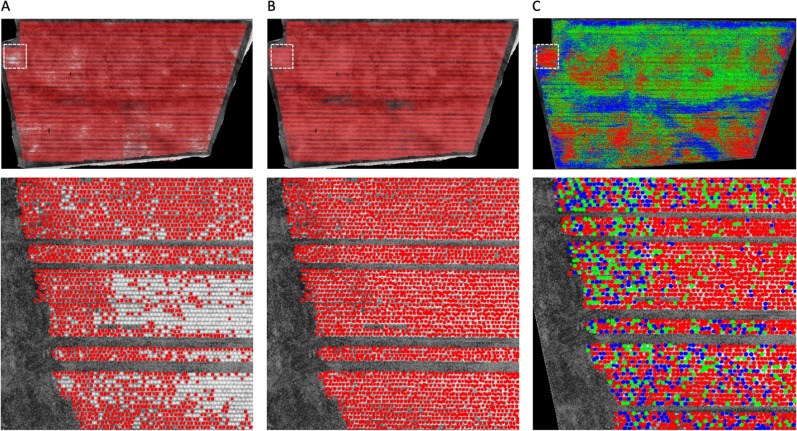
The improved results of the CNN model and the size classification of lettuce heads. **a** Wrongly detected lettuces in very bright regions and overly counted lettuces in very dark regions, in a one-million-head field. **b** Enhanced training datasets to retrain the model using the online-learning approach, which led to much better detection results. **c** A predefined colour code (small is coloured blue, medium is coloured green, and large is coloured red) is assigned to each recognised lettuce head across the field

After a CNN classifier was trained and the phenotypic analysis algorithms were implemented in the AirSurf-L, we used the software to recognise and classify lettuces in a series of ultra-large NDVI images. Initially, a broad range of sizes and orientations of lettuces with varying intensities were captured; however, the software failed to recognise lettuces in very bright regions and overly count lettuces in very dark regions (Fig. 5a), e.g., around 50,000 lettuces were wrongly detected in a one-million-head field (5% counting error). We found that this problem was caused by the trained CNN classifier, because a lettuce head is extremely tiny in an orthomosaic image (e.g., 11330 × 6600 pixels for a 7-hectare field when GSD is 3 cm, which contains over half million lettuces) under varied lighting conditions. To resolve this issue, we have designed a two-step solution: (1) sectioning the whole image into many 250 × 250 pixels sub-images, and (2) using a fix-sized bounding box (20 × 20 pixels) as a sliding window (with a stepping parameter of 5 pixels to reduce the computational complexity) to prune the detected lettuce objects in each 250 × 250 sub-image.

Another reason that caused the misdetection is due to overlapped lettuces as they could be detected repeatedly by the CNN classifier in a sub-image. Hence, we employed a non-maximum suppression (NMS) algorithm^30^ to rectify the detection. NMS uses probabilities to order the detected lettuce objects. After the 20 × 20 sliding window is performed and many small patches have been identified, the NMS algorithm computes an overlap coefficient to determine how to retain these patches. As lettuces are relatively well-spaced in the field, patches (i.e., bounding boxes) enclosing a complete lettuce signal are retained, whereas partially covered signals will be removed. To select the best overlap parameter computed by the NMS, a gradient descent method is formulated and explained in Supplementary File S3.

### Improved CNN classifier and the size categorisation

Besides the improved vision-based object detection, we also enhanced the training datasets by manually labelling an additional 500 lettuce signals within very bright or very dark regions. Then, newly labelled data was inserted into the training datasets to retrain the model through the online-learning approach^31^. The improved CNN model (see GitHub repository and Supplementary File S4) was tested on different experimental fields again and has dramatically enhanced the accuracy of lettuce detection (Fig. 5b).

Identified lettuces are individually analysed to determine their associated size category. The size classification is based on intensity and contrast values enclosed by the 20 × 20 bounding boxes, which is computed using the dot product of the histogram of pixel intensities and a weighted vector towards more pixel-based contrast values. The assumption of this design is that higher NDVI signals likely correlate with higher vegetation indices and hence bigger lettuce heads. The categorisation result of all lettuce heads is clustered into three size groups. Each lettuce is then coloured with a predefined colour code (Fig. 5c).

### A GPS-tagged harvest map

**Fig. 6:**
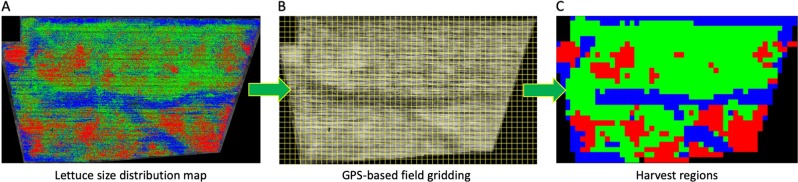
A GPS-based harvest map based on lettuce size classification. **a** A colour-coded lettuce size distribution map (small is coloured blue, medium is coloured green, and large is coloured red). **b** The field is segmented into thousands of grids based on the optimal GPS resolution. **c** Grids are coloured with the most representative lettuce size category across the image, representing harvest regions of the whole field

The final phase of the phenotypic analysis is to define harvest regions based on different sizes of lettuces. Using the size distribution map (Fig. 6a), the field is firstly segmented into many small grids based on the optimal GPS resolution determined by the altitude of the aerial imagery (3 cm GSD, in our case), as well as the size of the harvester machinery used by the grower. After dividing the field into thousands of grids (Fig. 6b), GPS coordinates of each grid are computed and each grid is then coloured with the most representative size category. By combining all coloured grids, a GPS-tagged harvest map is produced, representing harvest regions of the whole field (Fig. 6c). The harvest map can be used for designing harvesting strategies such as guiding a harvester to collect desired sized lettuces or arranging logistics based on the lettuce number and associated size counting. To facilitate precision agricultural practices, a result file (Supplementary File S2) is also generated by AirSurf-L at the end of the analysis workflow, containing information of each harvest grid, the associated GPS coordinates, lettuce size and number counting in each grid. To satisfy different needs for dissimilar requirements, the size of GPS-based harvest grids can be modified manually in the software.

### 3D visualisation for the harvesting strategy

**Fig. 7:**
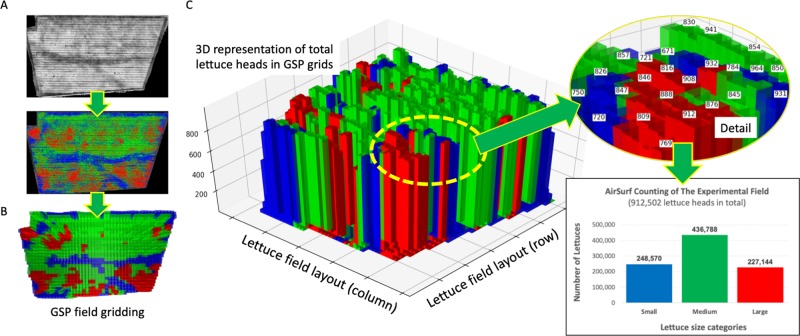
3D visualisation of lettuce harvest regions. **a** AirSurf-L reads an NDVI image and exports a lettuce size distribution map, where small lettuce is coloured blue, medium size is coloured green, and large lettuce is coloured red. **b** 3D visualising GPS-based field grids to present representative size categories. **c** A dynamic 3D bar chart is generated to present the relationship between lettuce number, infield layout, and the representative lettuce size, along with over lettuce number and size quantification

Figure 7 uses Python-based 3D Matplotlib library^32^ to show the GPS-tagged harvest map. When AirSurf-L reads an NDVI image, it computes the number of lettuce heads and associated size categories on the image (Fig. 7a). Then, by combining GPS-based field grids with the representative lettuce size in these grids (Fig. 7b), we produced a dynamic 3D bar chart script (Supplementary File S5) to present the lettuce number using the z axis, infield harvest regions (both columns and rows) using both x and y axes, and the representative lettuce size using the predefined colours (Fig. 7c). Through the 3D plot, users can zoom into any sub-region of the field to check lettuce number and representative size so that a precise harvesting strategy can be planned accordingly. The overall lettuce number and size counting of the experiment field can also be calculated.

### Validation of AirSurf-Lettuce

**Fig. 8:**
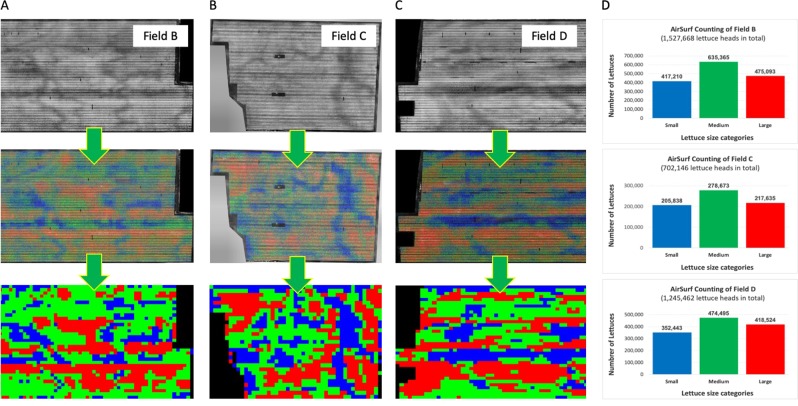
Applying AirSurf-Lettuce to count and classify millions of lettuce heads in three plantation fields across the Cambridgeshire, UK. **a–c** AirSurf-Lettuce is applied to count and classify millions of lettuce heads (small is coloured blue, medium is coloured green, and large is coloured red), in three plantation fields in the Cambridgeshire, UK. **d** The overall quantification of Lettuce heads and size categories in three fields

To verify AirSurf-L and the soundness the algorithm, we have applied the platform to count and classify lettuce heads in three unseen experimental fields in Cambridgeshire, UK (Fig. 8a–c). These fields contain between 700,000 and 1,500,000 lettuces and are located in different sites around the county. Traits such as the number of lettuces per field quantified by the platform (Fig. 8d) were compared with industrial estimates, showing a low error in lettuce counting (<5% difference). Besides the field-level comparison, we also randomly selected different sizes of subsections in an experiment field to evaluate AirSurf-L. We split these subsections into three sets (i.e., 36 small regions, 21 large regions, and 57 mixed regions), where the small regions have less than 400 lettuces, the large ones contain greater than 900 lettuces heads, and mixed regions contain a variety of lettuce heads. After that, laboratory technicians manually counted lettuce heads within these regions. The correlation between the manual and automated lettuce counting shows that, for the small regions, the correlation between the human and automatic counting is approximately 2% (R^2^ = 0.978); for the large regions, the value is around 0.8% (R^2^ = 0.988); and for mixed regions, the R^2^ correlation is over 0.9997. Supplementary Fig. 2 and Supplementary File S6 show the correlations between human and automatic counting for all three region groups.

## Discussion

Traditionally, measuring in-field crops on a large scale is very time-consuming and labour-intensive. It often requires destructive techniques, potentially error-prone manual counting, or estimates of traits that are key to yield production or crop quality^33^. Recent advances in ML/DL and CV techniques have led to an explosion of plant phenomics, which has rapidly improved our abilities in mining phenotypic information from large and complicated phenotyping datasets^34^. New data-driven analytic approaches are also changing plant phenomics – collecting big data (i.e., phenotyping) is no longer the bottleneck, instead how to extract biologically relevant information (i.e., phenotypic analysis) from big data has become the current challenge^35^. Hence, along with the development of aerial imaging and remote sensing technologies, it has become increasingly noticeable that the integration of scalable data collection, high-throughput phenotypic analysis, and yield predictive modelling is key to future crop research and precision agriculture^36^.

AirSurf-L introduced here has addressed a specific challenge in ultra-scale aerial phenotyping and precision agricultural management through combining aerial NDVI imagery, CV, ML/DL, and software engineering, with commercial lettuce production. The platform automates the quantification of millions of lettuces across the field, which allows us to connect research-based phenotypic analysis with real-world agriculture problems. As a cross-disciplinary project, we have chosen an agile R&D method, because technologies and requirements were constantly changing during the project. The results generated by AirSurf-L show a strong correlation between automatic counting and specialist scoring (*R*^2^ = 0.98). Hence, we are confident that AirSurf-L is capable of assisting fresh vegetable growers and farmers with their large-scale field phenotyping needs as well as yield-related trait analysis.

### Commercial impacts

Commercially, lettuce production offers an attractive economic profitability in comparison to many other Agri-Food businesses^37^. To date, lettuce businesses are worth billions of dollars and employ hundreds of thousands of permanent and seasonal workers globally. European vegetable growers alone produced 2.95 million tonnes of lettuce (and chicory) in 2016, a total annual value of €2.5 billion^38^. Further down the fresh produce supply chain, the planning and efficiency of many essential crop production activities are largely dependent on crop maturity date and the marketability of crops (i.e., the crop quality)^39^. Marketing activities such as logistics, trading, and product marketing need to be organised several weeks before the harvest; moreover, the booking and reservation of crop distribution, agricultural equipment, and associated commercial plans with retails also need to be determined beforehand^40^. By doing so, crop can be harvested at the right time, with maximised yield^41^. Our work contributes directly to lettuce production through improving the actual yield of lettuces and providing reliable quantification of crop quality (e.g., lettuce size), both of which are key factors for crop production, marketing, and supply chain management.

### Machine learning and computer vision in plant phenomics

Another aim of this work is to further ML- and CV-based software solutions in plant phenomics research. High-throughput plant phenotyping is a fast-growing research domain, covering many disciplines, from plant breeding, cultivation, remote sensing, to computing sciences^42^. The modular software development allows us to test and embed different open-source learning architectures^43^ (e.g., through the TensorFlow frameworks) and CV algorithms^44^ (e.g., Scikit-Image libraries) in AirSurf-L. Notably, it is worth pointing out that we have learned a number of lessons when applying ML/DL and CV in phenotypic analysis: (1) learning algorithms could perform badly if training datasets are not well-labelled and insufficient; (2) although ML/DL algorithms specialised in segmentation and classification if target objects are well-defined, there is still a big gap between object recognition and traits measurement; (3) meaningful phenotypic analysis not only requires sufficient biological understanding to define target traits in a logical manner, but also needs bespoke algorithms to engineer features so that traits can be quantitatively described. Hence, in plant phenomics research, biological questions, analytical solutions, and software implimentation need to be considered collectively in order to address approaching challenges.

### Limitations and further development of the platform

Besides the promising phenotypic analysis results presented in this article, there are still limitations of the platform need to be considered: (1) AirSurf-L has been tested with top-view iceberg lettuces mainly at H1 and H2 stages, which means that analysis error could increase if there are too many overlaps between lettuce heads, e.g., from H3 stage onwards. (2) As AirSurf-L has only been tested with NDVI imagery, it is important to add new functions to the platform to incorporate other vegetation indices acquired by multi- and hyper-spectrum imaging sensors. (3) As precision agriculture management decisions are normally based on imagery, soil and climate conditions, AirSurf’s results will be more reliable, if we could include soil information for each harvest region and field-level climate conditions. So, results can be compared between sites in multiple years. A potential approach is to incorporate ground-based phenotyping systems such as CropSight^45^ to feed environment data to the analysis. (4) The method was tested and validated in lettuce fields in a number of geographic locations following a standard aerial imaging procedure, data collected from different sites via varied aerial imaging strategies (e.g., different angles, altitudes and GSD) could improve the soundness and compatibility of the platform. (5) Key features were constructed by learning algorithms instead of engineered, which make learning models vulnerable when facing up to totally undefined datasets. Hence, ML/DL based phenotypic analysis algorithms need to update with new labelled training data for new crop species. (6) For a field of approximately one million lettuces, it takes about 4 h to analyse a field on a decent computer (2.5 GHz Intel Core i7, 8GB memory). Most of the computational time is occupied by the learning model to identify individual lettuces, which can be improved by parallel computing or GPU (graphic processing unit) processing to speed up the analysis.

### Prospects for crop research and precision agriculture

Together with recent advances in multi-scale remote sensing and phenotyping data management^45–47^, the platform could be relatively easily expanded to incorporate other crop species such as wheat and rice by retraining the learning model with additional datasets. By doing so, AirSurf could be developed into a more comprehensive analytic platform that will bring great significance to crop production and marketable yield for the Agri-Food sector. For example, the plant density of wheat and rice is closely related to the yield due to its influences on the allocation of water, light and fertilisers, which cannot be quantified using ground-based RGB imagery^48^. Hence, utilising the ultra-scale NDVI aerial imagery and related object recognition methods embedded in AirSurf-L, the platform is likely to benefit the assessment of sowing performance, emergence rate, and plant distribution. Then, through a multi-scale phenotyping approach (i.e. integrating ground-based workstations), breeders and crop researchers could make early predictions of the grain yield of crop genotypes in field experiments.

From a precision agriculture perspective, monitoring individual plant such as a lettuce head can enable accurate monitoring of crops during key growth stages across a plantation site. It can provide growers with the real number of crops in the field, based on which yield for harvest availability can be quantified instead of estimated. The calculation of crops can also lead to accurate agricultural inputs, facilitating automated variable-rate application of fertiliser, weed control, and pesticides through tractor software system with a more precise crop distribution map^49^. Furthermore, the close monitoring of key yield-related traits can be used to guide farmers and growers to reduce variability of agrichemical applications and irrigation in different fields, increasing harvest yield and better operating profit margin^50^. Finally, new analytic platforms such as the AirSurf-L platform shall largely embed in routine agricultural activities, so that no major extra costs are required, making new Agri-Tech solutions more adoptive by the Agri-Food sector.

## Conclusions

AirSurf-*Lettuce* automatically measures in-field iceberg lettuces using ultra-scale NDVI aerial images, with a focus on yield-related traits such as lettuce number, size categories, field size distribution, and GPS-tagged harvest regions. The analysis results are close to the manual counting and can be used to improve the actual yield. By monitoring millions of lettuces in the field, we demonstrate the significant value of AirSurf-L in ultra-scale field phenotyping, precise harvest strategies, and crop marketability before the harvest. We believe that our algorithm design, software implementation, the application of ML/DL and CV algorithms, and cross-disciplinary R&D activities will be highly valuable for future plant phenomics research that are destined to be more challenging. With continuous R&D work, we are confident that the platform has great potential to support the Agri-Food sector with a smart and precise crop surveillance approach of vegetable crops and therefore lead to better crop management decisions.

### Availability and requirements

Project name: AirSurf-Lettuce with G’s Growers

Project home page: https://github.com/Crop-Phenomics-Group/Airsurf-Lettuce

Source code: https://github.com/Crop-Phenomics-Group/AirSurf-Lettuce/

GUI software: https://github.com/Crop-Phenomics-Group/AirSurf-Lettuce/releases

Operating system(s): platform independent

Programming language: Python 3.6

Requirements: Keras, TensorFlow, Skimage, and Numpy.

License: BSD-3-Clause available at https://opensource.org/licenses/BSD-3-Clause

### Availability of supporting data

The datasets supporting the results presented here is available at https://github.com/Crop-Phenomics-Group/Airsurf-Lettuce/releases. Source code and other supporting data are also openly available in the GitHub repository.

## Supplementary information

Supplementary Figure 1.

Supplementary Figure 2.

Image calibration and pre-processing

Phenotypic analysis results

NMS algorithm

The CNN model

3D bar chart script

Human and AirSurf counting
