# Supplementary figures and images for: Combining computer vision and deep learning to enable ultra-scale aerial phenotyping and precision agriculture: A case study of lettuce production
Source: Hortic Res. 2019 Jun 1;6:70. doi: 10.1038/s41438-019-0151-5 (PMC6544649; doi:10.1038/s41438-019-0151-5)

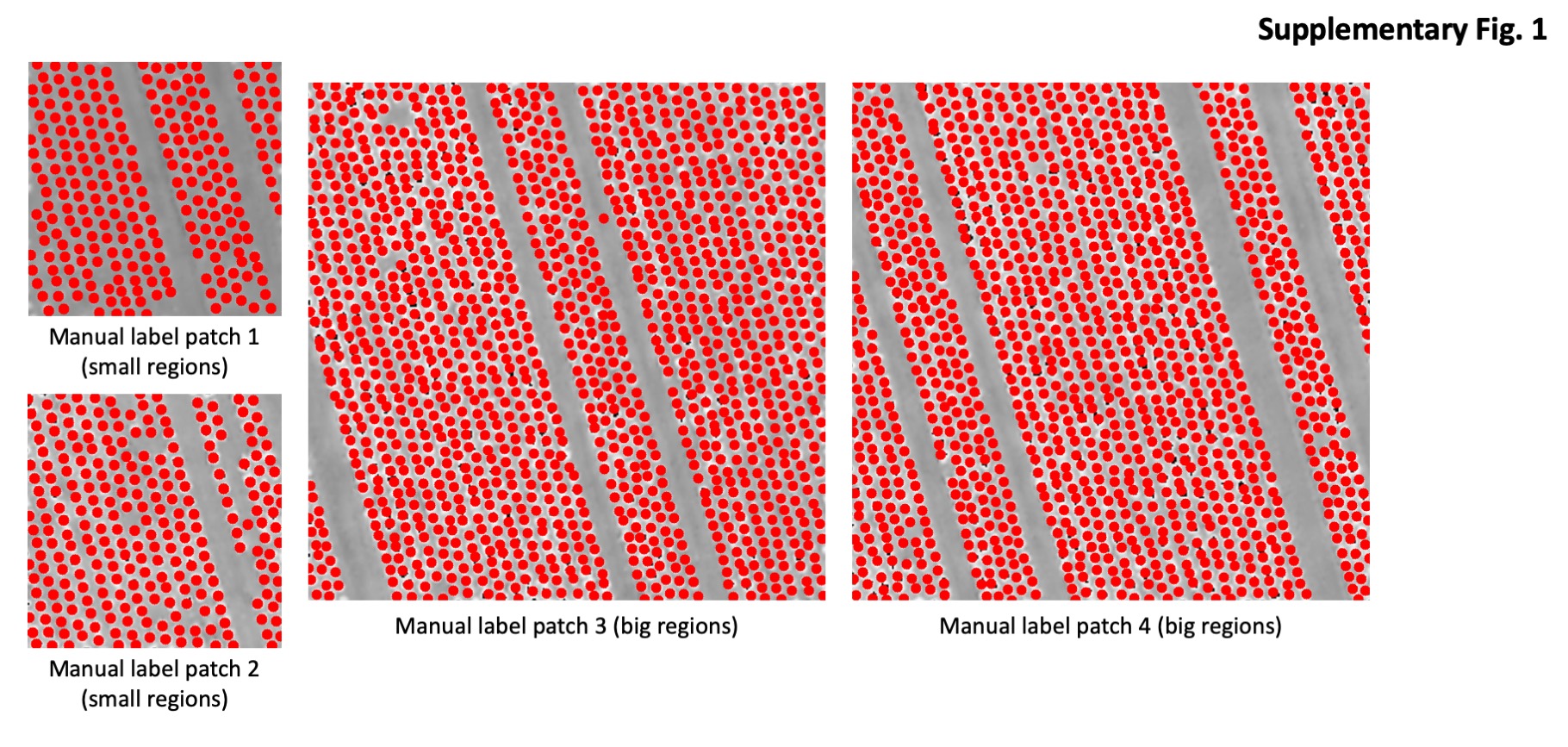

Supplement: Supplementary file 1 — Supplementary Figure 1. [file 41438_2019_151_MOESM1_ESM.jpg]

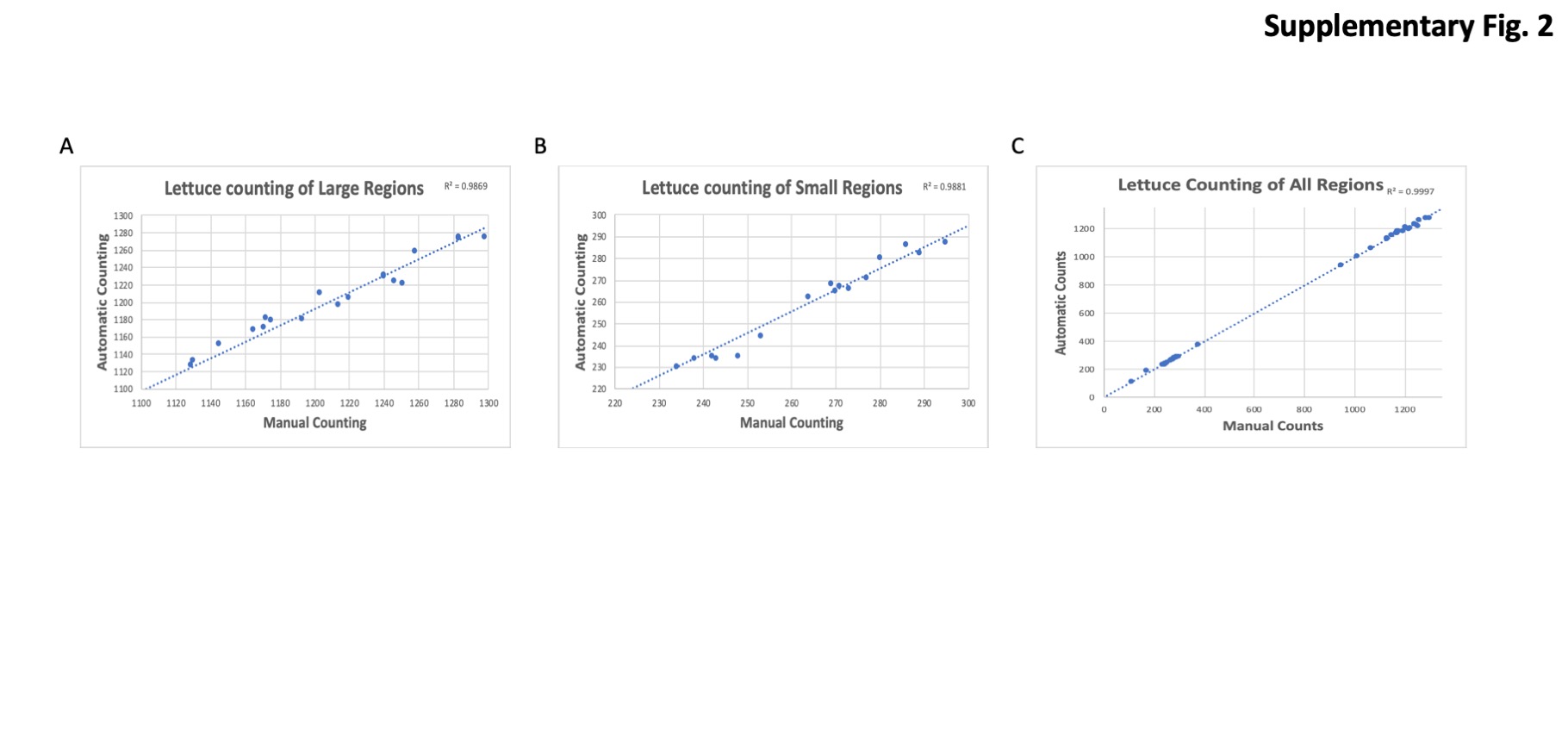

Supplement: Supplementary file 2 — Supplementary Figure 2. [file 41438_2019_151_MOESM2_ESM.jpg]
