# Supplementary material for: Combining computer vision and deep learning to enable ultra-scale aerial phenotyping and precision agriculture: A case study of lettuce production
Source: Hortic Res. 2019 Jun 1;6:70. doi: 10.1038/s41438-019-0151-5 (PMC6544649; doi:10.1038/s41438-019-0151-5)
Supplement: Supplementary file 3 — Image calibration and pre-processing [file 41438_2019_151_MOESM3_ESM.docx]

***Horticulture Research* Supporting Information 1**

Article title: **AirSurf-Lettuce: an aerial image analysis platform for ultra-scale lettuce phenotyping and precision agriculture using deep learning and computer vision**

For Contrast Limited Adaptive Histogram Equalization (CLAHE), we used the CLAHE in OpenCV (https://docs.opencv.org/3.1.0/d5/daf/tutorial_py_histogram_equalization.html) to implement our algorithm. Using CLAHE reduces the problem of homogeneous regions ending up with too much noise. With standard histogram equalization, images that only take up a small portion of the colour space often have noise amplified in regions of uniformity. Adaptive histogram equalization methods often perform standard histogram equalization in small regions of the images, which can again lead to noise amplification in some areas. CLAHE attempts to limit that by clipping and redistributing areas of high contrast across the entire region. The pseudo code can be seen as follows:

# create a CLAHE object (Arguments are optional).

clahe = cv2.createCLAHE(clipLimit=2.0, tileGridSize=(8,8))

# create an object to contain the input image as a numpy object

cl1 = clahe.apply(img)

CLAHE (img, clipLimit, tileGridSize):

for each region of tileGridSize in img:

perform Histogram Equalization in region to get contrasts

for contrasts over clipLimit:

redistribute values to all histogram bins
