# Supplementary material for: Combining computer vision and deep learning to enable ultra-scale aerial phenotyping and precision agriculture: A case study of lettuce production
Source: Hortic Res. 2019 Jun 1;6:70. doi: 10.1038/s41438-019-0151-5 (PMC6544649; doi:10.1038/s41438-019-0151-5)
Supplement: Supplementary file 5 — NMS algorithm [file 41438_2019_151_MOESM5_ESM.docx]

***Horticulture Research* Supporting Information 4**

Article title: **AirSurf-Lettuce: an aerial image analysis platform for ultra-scale lettuce phenotyping and precision agriculture using deep learning and computer vision**

Non-max suppression is a way of finding local maxima and then reducing all nearby values to 0. In our case, we take a group of potential boxes around a single lettuce and then return the box that we think best fits the lettuce while basically discarding the rest. This allows us to find the best (in this case defined as most likely) 20x20 box for each lettuce, and also prevents us from counting more lettuces than are in the field. Source code can be seen as follows:

# Malisiewicz et al.

def non_max_suppression_fast(boxes, probabilities, overlapThresh):

# if there are no boxes, return an empty list

if len(boxes) == 0:

return []

# if the bounding boxes integers, convert them to floats --

# this is important since we'll be doing a bunch of divisions

if boxes.dtype.kind == "i":

boxes = boxes.astype("float")

# initialize the list of picked indexes

pick = []

# grab the coordinates of the bounding boxes

x1 = boxes[:,0]

y1 = boxes[:,1]

x2 = boxes[:,2]

y2 = boxes[:,3]

# compute the area of the bounding boxes and sort the bounding

# boxes by the bottom-right y-coordinate of the bounding box

area = (x2 - x1 + 1) * (y2 - y1 + 1)

idxs = np.argsort(probabilities) # sort bounding box based on predictions.

# keep looping while some indexes still remain in the indexes

# list

while len(idxs) > 0:

# grab the last index in the indexes list and add the

# index value to the list of picked indexes

last = len(idxs) - 1

i = idxs[last]

pick.append(i)

# find the largest (x, y) coordinates for the start of

# the bounding box and the smallest (x, y) coordinates

# for the end of the bounding box

xx1 = np.maximum(x1[i], x1[idxs[:last]])

yy1 = np.maximum(y1[i], y1[idxs[:last]])

xx2 = np.minimum(x2[i], x2[idxs[:last]])

yy2 = np.minimum(y2[i], y2[idxs[:last]])

# compute the width and height of the bounding box

w = np.maximum(0, xx2 - xx1 + 1)

h = np.maximum(0, yy2 - yy1 + 1)

# compute the ratio of overlap

overlap = (w * h) / area[idxs[:last]]

# delete all indexes from the index list that have

idxs = np.delete(idxs, np.concatenate(([last],

np.where(overlap > overlapThresh)[0])))

# return only the bounding boxes that were picked using the

# integer data type

return boxes[pick].astype("int"), probabilities[pick]
